# Rhinitis, sinusitis and ocular disease – 2091. The MSYPQ: repeatability and applicability to rhinitis/rhinosinusitis

**DOI:** 10.1186/1939-4551-6-S1-P169

**Published:** 2013-04-23

**Authors:** Amtul Salam Sami, Glenis Scadding, Masood Malik, Mohammed Amjad

**Affiliations:** 1 Ent and Allergy Department, London, UK; 2 Services Hospital, Ent Department, Pakistan

## Background

Sinonasal disease is significantly prevalent in the adult community, this research explores its prevalence within the 11-16 age group in London, UK and in Lahore, Pakistan. Epidemiological assessment was accompanied by assessment of associated compliactions.

The Sino-Nasal Outcome Test -20 (SNOT-20) questionnaire, a disease related quality of life instrument, was modified for use in secondary school children for this project. The Modified SNOT-20 for Young Person Questionnaire (MSYPQ) was used in secondary school children in East London with the result compared to a similar adult survey. An analysis of the repeatability of the MSYPQ was carried out in a sample population of 11-16 years olds from Lahore, Pakistan.

## Methods

The pilot project analysed MSYPQ according to and deemed concurent with EPOS criteria, this confirmed that MSYPQ identifies disease subjects and is a good instrument to assess the effect on quality of life.

The MSYPQ was used in three large East London schools with analysis for disease prevalence, associated symptoms and effects on quality of life. A further comparative study was designed whereby 200 subjects from a secondary school sample (randomly selected and between 11-16 years old) in Lahore, Pakistan were analysed using MSYPQ to assess the prevalence of rhinitis/rhinosinusitis and its associated comorbidities.

## Results

The results showed that over 32% of secondary school children in the London sample suffered rhinitic/rhinosinusitis symptoms (cough was the most significant symptom). A similar prevalence of 30% was found in adults. More than 21% had their quality of life affected by their symptoms and more than 47% took between 2-15 days off school due to symptoms, compared to 25% and 10% respectively in adults. Data analysis from Lahore confirmed that over 25% suffer from rhinitis/rhinosinusitis with significant associated comorbidities. Its impact on their quality of life and educational performance is substantial.

## Conclusions

This analysis confirmed that sinonasal disease is a common problem in the 11-16 year age group; it impacts on quality of life and performances at school. This study also confirms that MSYPQ is a reliable and repeatable tool for identifying the prevalence and impact of rhinitis/rhinosinusitis.

